# Analysis of spatial and temporal changes and driving forces of arable land in the Weibei dry plateau region in China

**DOI:** 10.1038/s41598-023-43822-3

**Published:** 2023-11-23

**Authors:** Panpan Zhang, Liheng Xia, Zenghui Sun, Tingyu Zhang

**Affiliations:** 1 Institute of Land Engineering and Technology, Shaanxi Provincial Land Engineering Construction Group Co., Ltd, Xi’an, China; 2 Shaanxi Provincial Land Engineering Construction Group Co., Ltd, Xi’an, China; 3 Technology Innovation Center for Land Engineering and Human Settlements, Shaanxi Land Engineering Construction Group Co., Ltd and Xi’an Jiaotong University, Xi’an, China; 4 Key Laboratory of Degraded and Unused Land Consolidation Engineering, Ministry of Natural Resources, Xi’an, China

**Keywords:** Environmental sciences, Agroecology

## Abstract

Arable land is the lifeblood of food production, it is of great significance to promote the protection of arable land and ensure national food security by accurately understanding the change law of cultivated land and its driving mechanism. This study takes the Weibei dry plateau region of China as an example, explores its spatial and temporal change characteristics through the center of gravity shift and land use shift matrix, and couples the geographic probe model to reveal the driving mechanisms affecting arable land change. The results show that in the past 25 years, the total arable land area in the Weibei Dry Plateau Region of China has decreased by 5.58%, and the stability of arable land resources in the whole region has weakened. The center of gravity of arable land shifts to the northeast, and the standard deviation ellipse of arable land mainly undergoes the change process of "increase (1995–2015)-decrease (2015–2020)", and the spatial distribution of arable land tends to be dispersed. In the LISA frequency mapping, the proportion of stable constant and low-frequency areas is as high as 89.58%, and the spatial pattern of cultivated land is relatively stable. Medium and high frequency areas. The transformation mode is mainly "low-low" aggregation, "low–high" aggregation is not significant, and the decline of cultivated land in the study area is more obvious. In the past 25 years, a total of 1017.26 km^2^ of arable land was converted to construction land. The explanatory power of the influencing factors varies in each period (0.299 to 0.731), with total agricultural machinery power has the strongest explanatory power of 0.694, 0.592, and 0.731, respectively. The interaction between slope and annual average temperature and other factors being the highest, both greater than 0.8. Through the construction of LISA frequency mapping, combined with the center of gravity model and standard deviation ellipse, the spatial evolution trend of regional arable land is more comprehensively and dynamically grasped. By using the geodetector model, the driving mechanism of the changes of arable land is revealed comprehensively, which provides a theoretical basis for the scientific management and effective protection of arable land resources and a basis for decision-making.

## Introduction

Arable land is the essence of land resources, and is the foundation and main carrier for the development of agricultural production activities^1–3^. Since 1978, a large area of high-quality arable land has been occupied in the process of urban expansion and industrial development, resulting in the increasing fragmentation of contiguous arable land and the destruction of arable land ecosystems, which has seriously restricted the sustainable development of arable land resources^4–6^. Over the few decades, arable land resources in different regions of China have shown a decreasing trend. In recent years, arable land resources in different regions of China have shown a decreasing trend.,and it in Nanjing metropolitan area decreased by 57.24% from 1985 to 2010 on the whole; from 2000 to 2018 alone, the area of arable land in the karst mountainous area of Guizhou turned into forest land, grassland and construction land by up to 121,900 hm^2^. With the change of urban scale, extensional expansion and complex population composition, arable land around cities is constantly being eroded and cut, and the fragmentation of arable land is deepening^7–9^. For example, the landscape fragmentation index of arable patches in Horqin Sandy Land of China increased from 2.82 in 1980 to 6.53 in 2010. In addition, the ecological degradation caused by the change of arable land has become a prominent issue. From 2009–2015, the ecological deficit of cropland use in China increased from 2.04 × 107 hm^2^ to 4.29 × 107 hm^2^; 166.82 billion in global ecological service value was lost due to cropland change from 1992 to 2015^10^. Weibei Dry Plateau Region in Shaanxi Province is located in the southern part of the Loess Plateau hilly and ravine area and the northern part of the Guanzhong Plain, which is the main base of agricultural production in the northwest region. Due to the long-term pursuit of economic benefits, the unreasonable utilization of arable land resources has resulted in an overall decline in quality^11^. Therefore, exploring the change of arable land and its driving forces is of great practical significance for the protection and sustainable utilization of arable land resources^12,13^.

Utilization of arable land resources has received considerable attention in China in recent years. Research has focused on the spatial distribution of arable land, optimization of farmland space, the driving mechanism of arable land change, and the prediction of arable land change^14–16^. Due to the abundant light and heat resources in the Weibei Dry Plateau Region of Shaanxi Province, previous studies have paid more attention to the utilization and evaluation of the forest and fruit industry, but there is little systematic research on the spatiotemporal evolution and driving forces of arable land in this region. However, most of the researches mainly reveal the spatial distribution of arable land at the macro level, and there are fewer researches on the spatial correlation of arable land, and lack of dynamic revelation of the changing law of the spatial pattern of arable land. The spatial autocorrelation method can well reflect the distribution characteristics of spatial variables and the degree of influence on the neighboring areas, but the existing research mostly focuses on the analysis of the spatial aggregation characteristics of a certain period of time, and there are fewer studies on the integration of spatial correlation under multi-temporal scales. In this paper, based on the geographic information mapping model, we match the "spatial aggregation type map" with the "time-varying process spectrum" to construct the LISA frequency map, which dynamically demonstrates the spatial aggregation state under multi-temporal scales and intuitively reflects the variability of the spatial autocorrelation changes and trends. The center of gravity model and the standard deviation ellipse are introduced to dynamically reflect the evolution process and trajectory of the spatial pattern of arable land, and intuitively reveal the magnitude and trend of the spatial change of arable land^17^.

The Geodetctor is a good spatial statistics method for measuring and attributing spatial stratified heterogeneity, which has been widely used in the fields of land use, regional planning, ecology, environment and remote sensing^18^. The key principle of its model is that it can not only determine the strength of the effect of single-factor data by q-value, but also analyze the strength of the interaction of two factors, which can explain the driving mechanism of arable land change more comprehensively^19^. Therefore, this study integrates GIS tools and Geodetector to systematically depict the spatial and temporal characteristics of arable land in the Weibei Dry Plateau Region of Shaanxi Province over the past 25 years, and to explore its driving factors impacting spatiotemporal evolution. It is expected to provide a scientific basis for arable land conservation and agricultural policy formulation. The research results are of reference value for the conservation of arable land resources in the Weibei Dry Plateau Region and the protection of national food security^20–24^.

## Materials and methods

### Study area

**Figure 1:**
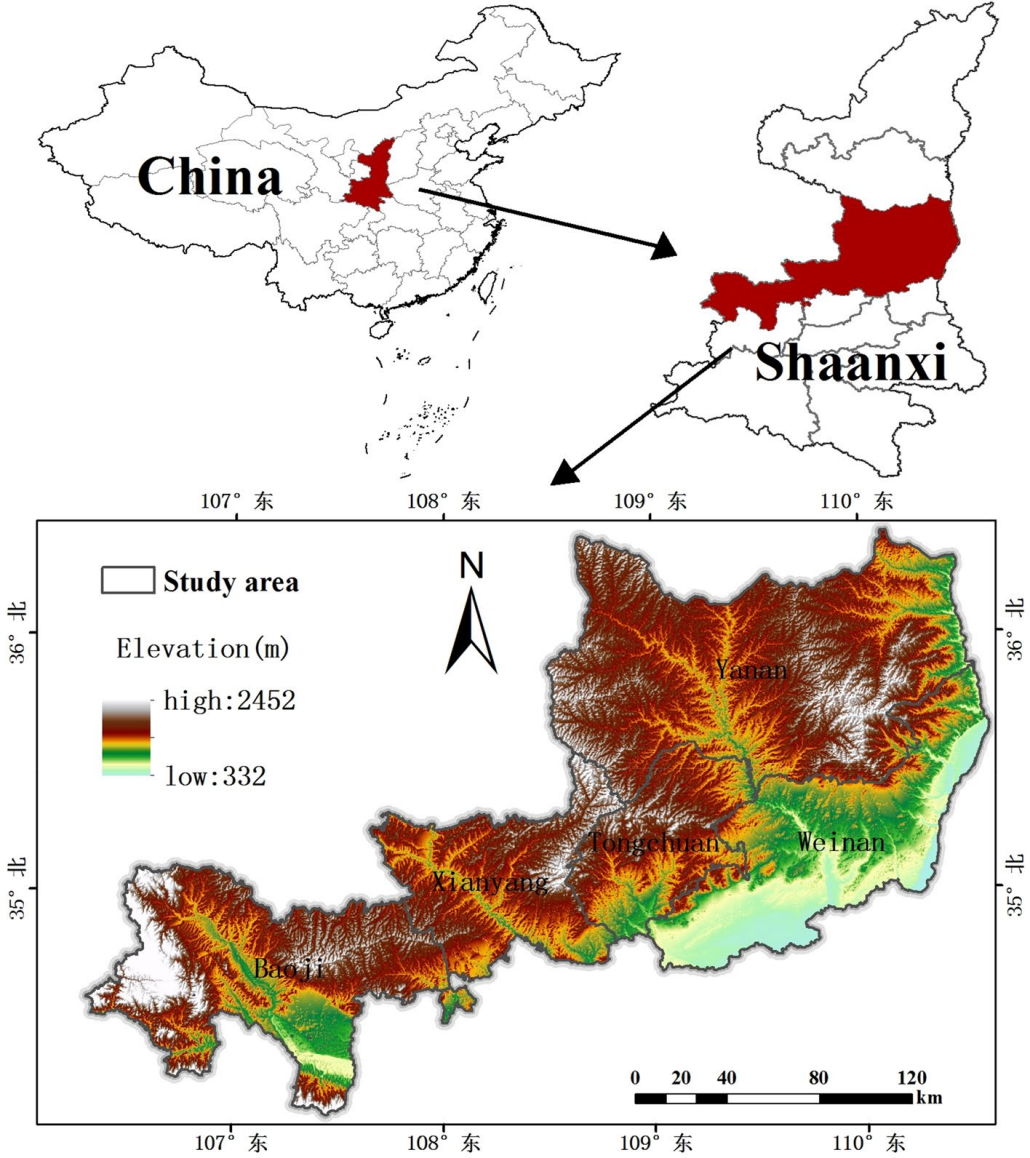
Weibei dry plateau region in Shaanxi Province.

The Weibei Dry Plateau Region (34°22'–36°14' N, 106°29'–110°36' E) in Shaanxi Province is located in central China, including 25 districts and counties in 5 cities: Baoji, Xianyang, Weinan, Yan'an, and Tongchuan (Fig. 1), with a total land area of about 39,600 km^2^ and a total arable land (including garden land) of about 12,500 km^2^. The elevation of the study area ranges from 331 to 2452 m above sea level. The landscape features are mainly plateau gullies, and the area of gullies accounts for more than 55% of the total area. The average annual temperature ranges from 8.6 to 13.5 °C, and the annual rainfall ranges from 520 to 650 mm, with most of the precipitation falling in July and September. Agriculture in Weibei Dry Plateau Region mainly relies on natural precipitation, and dry land accounts for about 75% of the total arable land area.

### Data sources

The remote sensing data of each period are obtained from NASA Landsat data, including Landsat-5 TM images in 1995, 2000, 2005, 2010 and 2015 and Landsat-8 OLI images in 2020, where Landsat-5 images have a resolution of 30 m × 30 m and Landsat-8 images The resolution of Landsat-5 image is 30 m × 30 m and Landsat-8 image is 15 m × 15 m. The images are acquired in May–August and the cloudiness is less than 5% in each year, and the orbital numbers are 126/35, 126/36, 127/35, 127/36, 128/36, and the images are pre-processed by radiometric calibration, FLAASH atmospheric correction, image mosaic, cropping and fusion. The croplands were decoded in ENVI software by Classification supervised classification for each period (Attachment 1). The results were calculated by confusion matrix, and the overall accuracy of classification reached 90.76% with Kappa coefficient of 0.8814.

The DEM data of the study area were collected from National Aeronautics and Space Administration (NASA, https://earthdata.nasa.gov/) with a spatial resolution of 30 m × 30 m. Landform type, soil type, temperature, precipitation and nighttime lighting data were obtained from the Resource and Environment Science Data Center of the Chinese Academy of Sciences (http://www.resdc.cn/) with a spatial resolution of 1 km × 1 km.

The socioeconomic data of the counties in the study area were mainly derived from Shaanxi Statistical Yearbook (2000–2020), China County Statistical Yearbook (2000–2020) and Shaanxi Regional Statistical Yearbook (2000–2020), including the total population, population density, total agricultural machinery power, total grain production, added value of primary industry, added value of secondary industry, GDP per capita, and fixed asset investment of the study area in the three periods.

### Research methods

#### Net rate of change of cropland

The net change rate of cropland is usually used to reflect the extent of the inflow or outflow of cropland during a certain period. It is generally expressed as the ratio of the net change of cropland during the study period to the total amount of cropland at the beginning of the study period. The calculation formula is as follows:

1 $$N = \frac{{U_{b} - U_{a} }}{S} \times 100\%$$

where N is the net rate of change of cropland; S is the total area of cropland at the beginning of the period in the study area; U_b_ and U_a_ are the increase and decrease of cropland during the study period, respectively^25^.

#### Dynamic attitude of cropland change

The change of dynamics degree arable land refers to the change of arable land in a certain period of time, which is used to express the stability of arable land resources, the higher the dynamics degree, the worse the stability of arable land resources, and the calculation formula is:

2 $$V = \frac{{\Delta U_{i} }}{t \cdot S} \times 100\%$$

where V is the dynamic attitude of cropland change; ∆U_i_ is the amount of change in cropland area during the study period, S is the total area of cropland at the beginning of the study area, and t is the length of the study interval^26^.

#### Center of gravity

Arable land Center of gravity is mostly used to study the process of spatial location change of a geographical element in the process of regional development. The model reflects the changing trends of spatial elements through the direction, distance and speed of center of gravity migration. This paper uses the center of gravity model to reveal the spatial aggregation characteristics and change trends of arable land. The calculation formula is:

3 $$\overline{X} = \mathop \sum \limits_{i = 1}^{n} M_{i} X_{i} /\mathop \sum \limits_{i = 1}^{n} M_{i}$$

4 $$\overline{Y} = \mathop \sum \limits_{i = 1}^{n} M_{i} X_{i} /\mathop \sum \limits_{i = 1}^{n} M_{i}$$

where ${\overline{\text{X}}}$, ${\overline{\text{Y}}}$ are the center of gravity coordinates at the beginning of the study; M_i_ is the arable land area of the i-th township, km^2^; X_i_ and Y_i_ denote the center of gravity coordinates of the i-th township.

The standard deviation ellipse can visually reflect the aggregation status and offset trend of arable land, and is mainly composed of the rotation angle θ, the standard deviation along the major axis (long axis) and the standard deviation along the minor axis (short axis). The long half-axis of the ellipse indicates the direction of the distribution of arable land, and the short half-axis indicates the extent of the distribution of arable land. In this study, based on the center of gravity model, the standard deviation ellipse s are constructed to reflect the spatial pattern of arable land.

#### Land use transfer matrix

The land use transfer matrix can fully and specifically describe the structural characteristics of regional land use change, and can reflect the direction of land use change guided by human activities. The general form of land use transfer matrix is as follows:

5 $$S_{ij} = \left[ {\begin{array}{*{20}c} {S_{11} } & {S_{12} } & \ldots & {S_{1n} } \\ {S_{21} } & {S_{22} } & \ldots & {S_{2n} } \\ \ldots & \ldots & \ldots & \ldots \\ {S_{n1} } & {S_{n2} } & \ldots & {S_{nn} } \\ \end{array} } \right]$$

where i and j represent the land use types before and after the transfer, respectively; n is the number of land use types before and after the transfer; s_ij_ stands for the area of the land use type of i converted to the land use type of j.

#### Geodetector

Geodetector is a spatial statistical method for detecting spatial heterogeneity and quantifying the influence of factors. In this study, factor detector and interaction detector were used to explore the single-factor influence and interaction of drivers of arable land change in the Weibei Dry Plateau Region.

#### Factor detector

Factor detector detects the extent to which the influence factor X explains the spatial differentiation of arable land in the Weibei Dry Plateau Region. The explanatory power of one factor is measured by the q-value:

6 $$q = 1 - \frac{1}{{N\partial^{2} }}\mathop \sum \limits_{i = 1}^{L} N_{i} \partial_{i}^{2}$$

where N_i_ and N are the number of units in class i and the whole region, respectively; L is the stratification of the indicators affecting arable land change; ∂_i^2^ and ∂^2^ are the variance of each factor category and the whole area Y. The q value ranges from 0 to 1, and the larger the value of q, the stronger the explanatory power of the potential factor X on the explanatory variable arable area Y, and vice versa.

#### Interaction detector

Interaction detector identifies the interaction between natural and socioeconomic factors. It is able to assess whether the explanatory power of the joint effect of the two factors on the arable area is enhanced, diminished or independent of each other. First, q values of the two factors X1 and X2 of Y are calculated separately, then q values of their interactions are calculated, and finally q(X1), q(X2), q(X1 ∩ X2) are compared and their interactions are judged^27–29^.

### Statement

The use of plants in the present study complies with international, national and/or institutional guidelines.

## Results and analysis

### Analysis of total arable land changes from 1995 to 2020

#### Characteristics of the overall change in the amount of arable land

**Figure 2:**
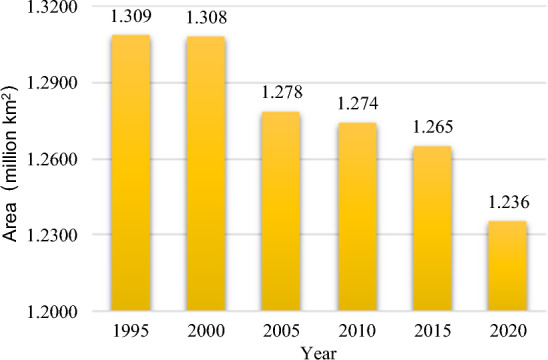
Changes in the area of arable land in the study area from 1995 to 2020.

We observed a decreasing trend of the arable land area in the study area from 1995 to 2020 (Fig. 2). Compared with 1995, the arable land area in the study area decreased by 5.58% in 2020. Specifically, the loss of arable land was more serious in two periods during 2000–2005 and 2015–2020, and the arable area decreased by 294.74 km^2^ and 293.96 km^2^, respectively.

#### Characteristics of net change rate of arable land

**Figure 3:**
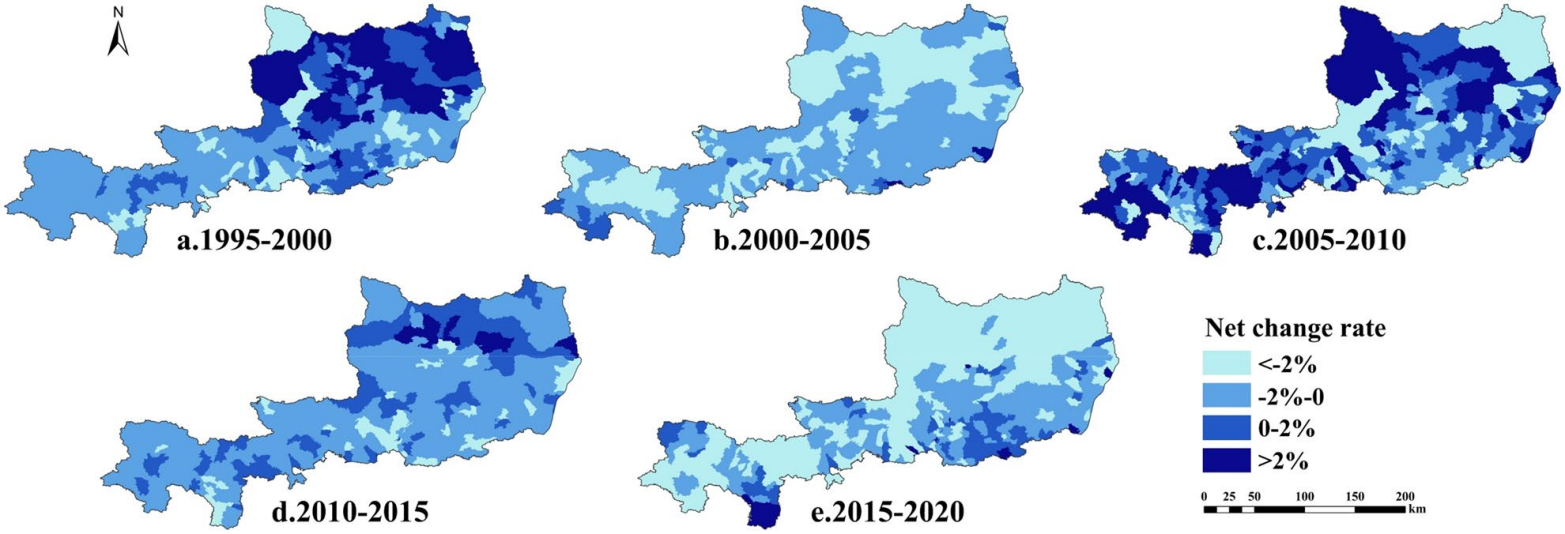
Spatial distribution of net change rate of arable land in the study area from 1995 to 2020.

Figure 3 shows that the inflow and outflow of cropland in the study area were basically the same between 1995–2000 and 2005–2010, and showed an increase in the east and a decrease in the west. In contrast, most of the rest of the time was in a net loss of cropland, and the loss was more serious in 2000–2005 and 2015–2020, with the net loss of cropland exceeding 2% in most areas, mainly in the northern part of the study area. In general, the net loss of cropland in the study area has been increasing over the past 25 years, and the loss of cropland in the northern plateau area is more serious.

#### Characteristics of the dynamics degree of arable land

Since 1995, except for the southern part of the study area, the dynamic degree of arable land change in most areas has exceeded 6%, and the change of arable land in the study area has been relatively active. Only during 2010–2015, the dynamic degree of arable land was low, and remained within 2% in most areas. From 1995 to 2020, the dynamic degree of arable land showed the characteristics of high in the north and low in the south. The dynamic degree of most of the northern areas was greater than 8%, and the change of arable land was very active. Overall, the dynamic degree of arable land change in the study area from 1995 to 2020 remained at a moderate level, and the stability of arable land resources in the whole region was weakened, and the stability of arable land resources in the northern plateau area was poor, which was nearly the same with the characteristics of the net change rate of arable land.

### Analysis of spatial change characteristics of arable land

#### Center of gravity evolution analysis of arable land

**Figure 4:**
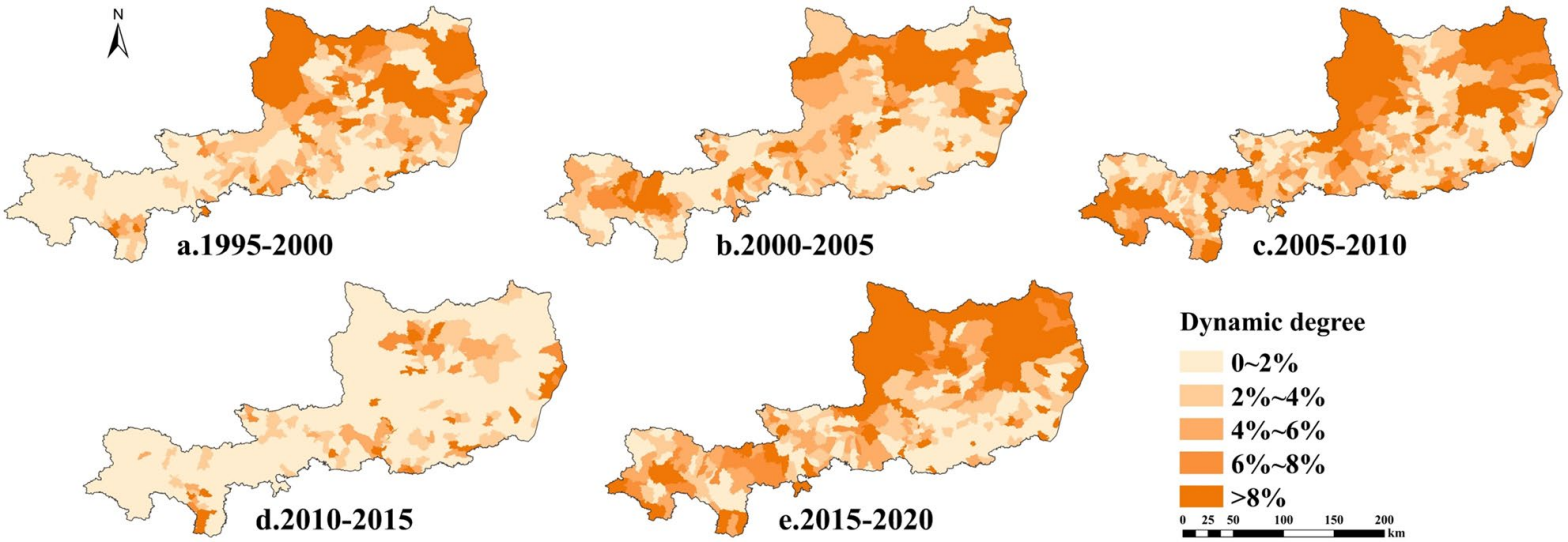
Change of the dynamics degree of arable land in the study area from 1995 to 2020.

**Figure 5:**
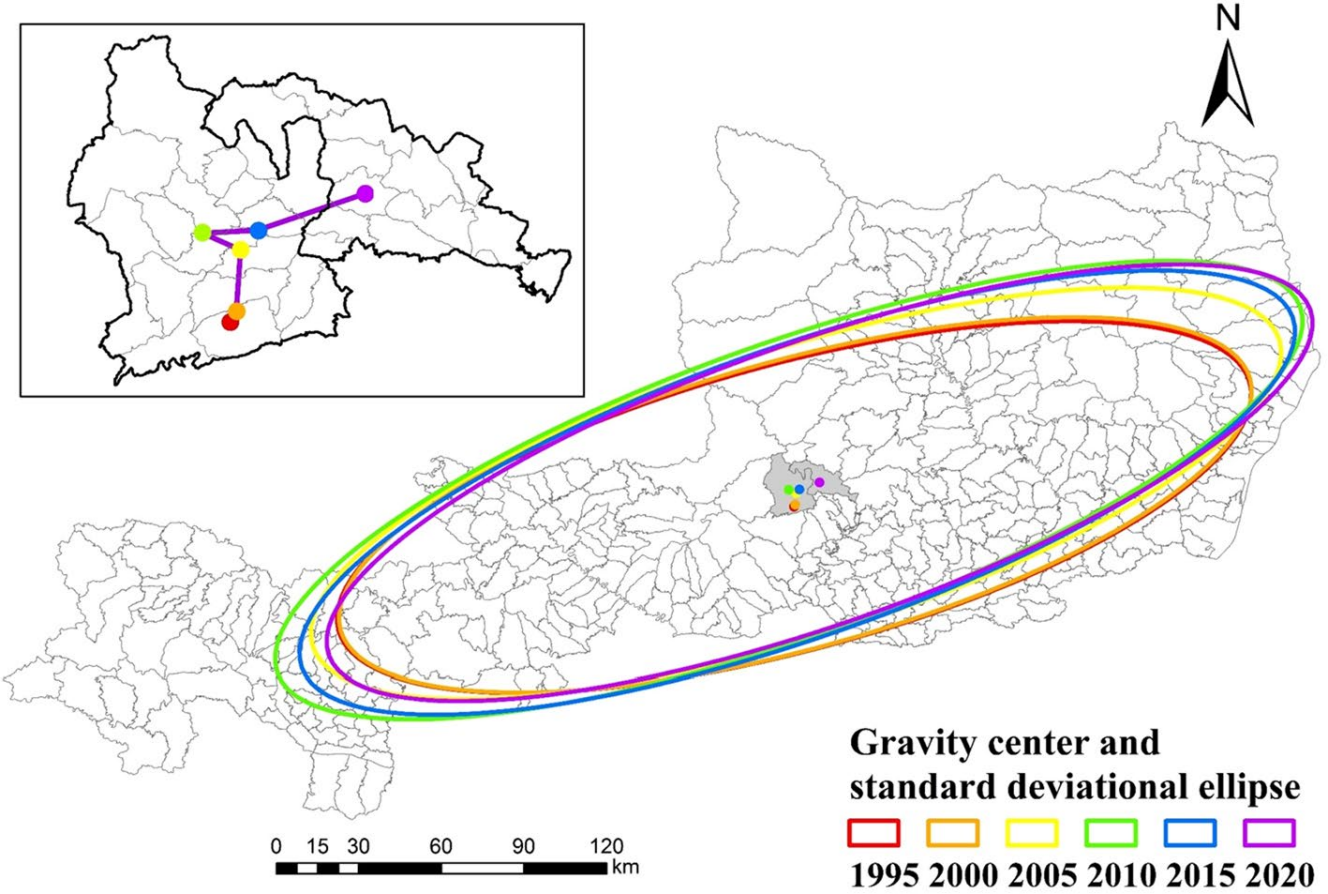
Distribution of arable land centers and standard deviational ellipses of arable land of the study area from 1995 to 2020.

Using the arable land center of gravity model to obtain the migration direction, distance and speed of the proportion of arable land for each period. The results are shown in Figs. 4, 5 and Table 1. The change of arable land in the study area is mainly divided into the following two stages:

**Table 1:** Shift of arable land center of gravity in the study area during 1995–2020.

Shift of gravity center	Period				
	1995–2000	2000–2005	2005–2010	2010–2015	2015–2020
Moving direction	Northeast	Northeast	Northwest	North	Northeast
Movement distance (m)	826.70	4223.50	2447.18	3144.58	6518.88
Movement speed (m·a^-1^)	165.34	844.70	489.44	628.92	1303.78

From 1995 to 2000, the center of gravity of arable land changed in Jinyuan Village, Yaoqu Town. The overall movement of arable land center of gravity was oriented in the north–east at a speed of 165.34 m·a^-1^. In the mid-1990s, limited by factors such as water resources, infrastructure construction, and farming technology, the agricultural economy developed at a low rate and urban expansion was slow. Therefore, the center of gravity of arable land moved less and slowly.

From 2000 to 2020, due to the government has increased investment in agricultural modernization, agricultural infrastructure was gradually improved and updated, and the penetration rate of new agricultural technologies increased, which led to the rapid development of agriculture in the northern mountainous areas. In addition, coupled with the expansion of southern towns occupying arable land, which presented an overall movement to the northeast in the plateau gully area. Variations in the distance and rate of cropland center of gravity movement increased from 2000 to 2015, with the largest offset distance of 6518.88 m between 2015 and 2020, indicating that the decrease of cropland in the south and increase of cropland in the north during this period was relatively large. The possibility of this phenomenon is that arable land in the south has decreased more due to rapid economic development and the continuous expansion of towns. In comparison, the implementation of arable land conservation projects such as ditch management and land creation in Yan'an City in the north has led to an increase in arable land. Therefore, the offset distance is larger under the dual effect of north and south.

In conclusion, the center of gravity of cropland in the study area has generally shifted to the northeast over the past 25 years, with a distance of 17,160.84 m. The rate of shifting has shown an overall increasing trend. the rate of shift of the center of cropland increased from the initial 165.34 m a^-1^ to 1303.78 m a^-1^ in 25 years, and movement speed showed an increasing trend.

#### Standard deviational ellipse model of arable land

The parameter changes of the standard deviation ellipses of arable land are shown in Table 2, and the spatial distribution is shown in Fig. 5. It can be seen that the standard deviational ellipse change in the study area has a certain directionality, which is correlated with the shift of the center of gravity of arable land. From 1995 to 2000, the rotation angle narrowed from 62.26 to 61.76°, and the the distribution showed a movement to the northeast. The rotation angle did not change much during 2000 to 2020, increased and had a trend of shifting to the northeast, and the spatial distribution of the northeast direction kept increasing.

**Table 2:** Standard deviational ellipses of arable land of the study area during 1995–2020.

Year	1995	2000	2005	2010	2015	2020
Rotation (°)	62.26	61.76	61.77	61.64	61.86	61.91
Standard deviation along the x-axis (km)	164.09	164.30	164.69	166.67	165.43	164.43
Standard deviation along the y-axis (km)	46.65	47.84	48.97	48.45	48.81	48.67
Ellipse area( km^2^)	24,040.24	24,688.74	25,328.31	25,361.07	25,362.21	25,135.18

The spatial distribution pattern of the standard deviation ellipse is consistent with the distribution direction of cropland in the study area, and basically covers the dense cropland area from west to east, which is the central part of Baoji City, the central part of Xianyang City, and Weinan City to Fuping County and Pucheng County, respectively. The area of the standard deviation ellipse increased by 1904.93 km^2^, and the area of the standard deviation ellipse mainly showed an "increase (1995–2015)-decrease (2015–2020)" pattern of change, indicating that the spatial distribution of cropland in the study area gradually tends to be dispersed, and the rate of dispersal is decreasing.

### LISA frequency geo-spectrum

#### Local space autocorrelation

**Figure 6:**
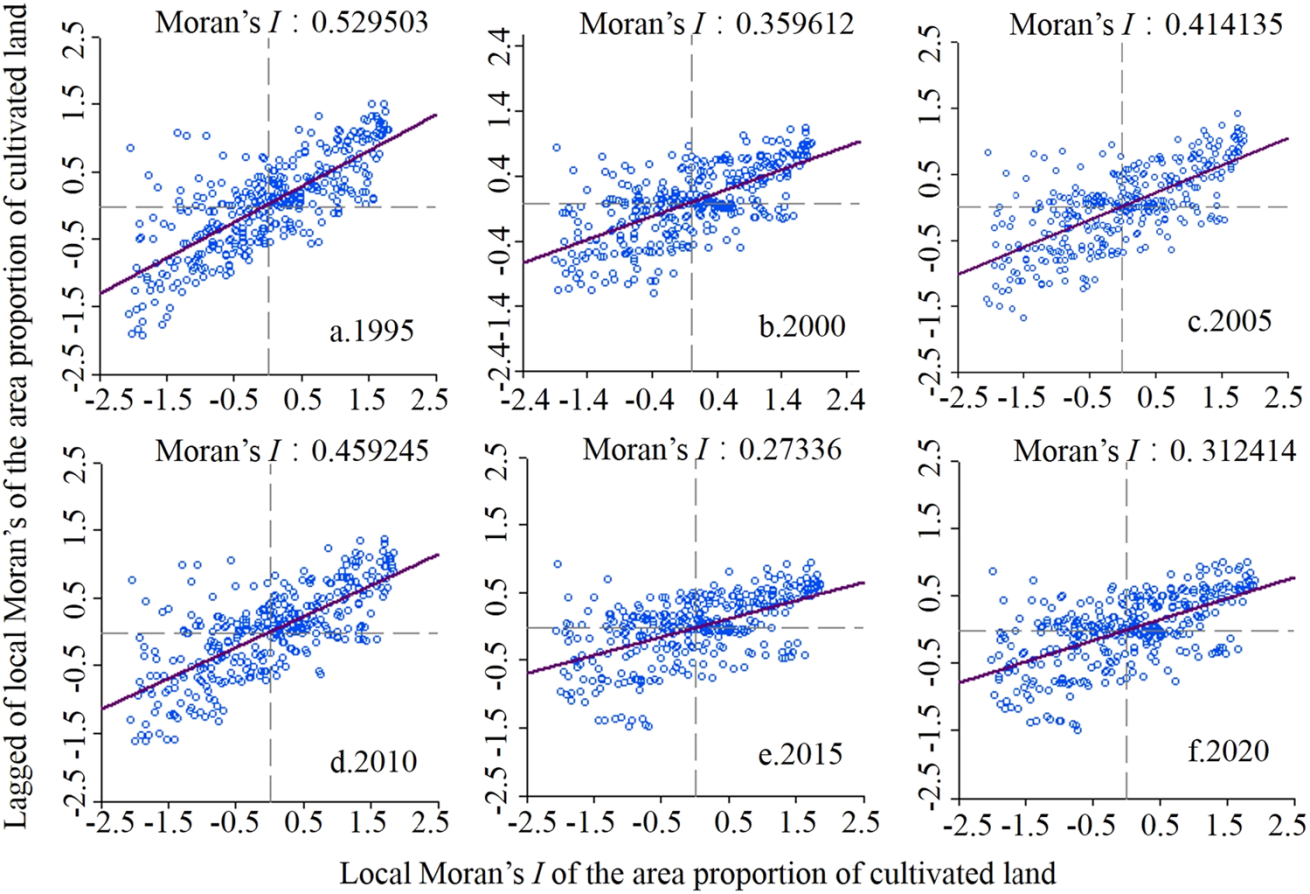
Scatter-plots of local Moran’s I for the areal proportion of arable land in the study area from 1995 to 2020.

As can be seen in Fig. 6, the local Moran's I indices for the proportion of arable land in the townships of the study area were all significantly different, ranging from 0.273 to 0.529. Most of the townships are located in quadrants 1 and 3, which belong to the "high-high" and "low-low" types respectively, i.e., the area ratio of the region shows high or low values of aggregation. Most of the arable land in the study area was distributed in clusters.

**Figure 7:**
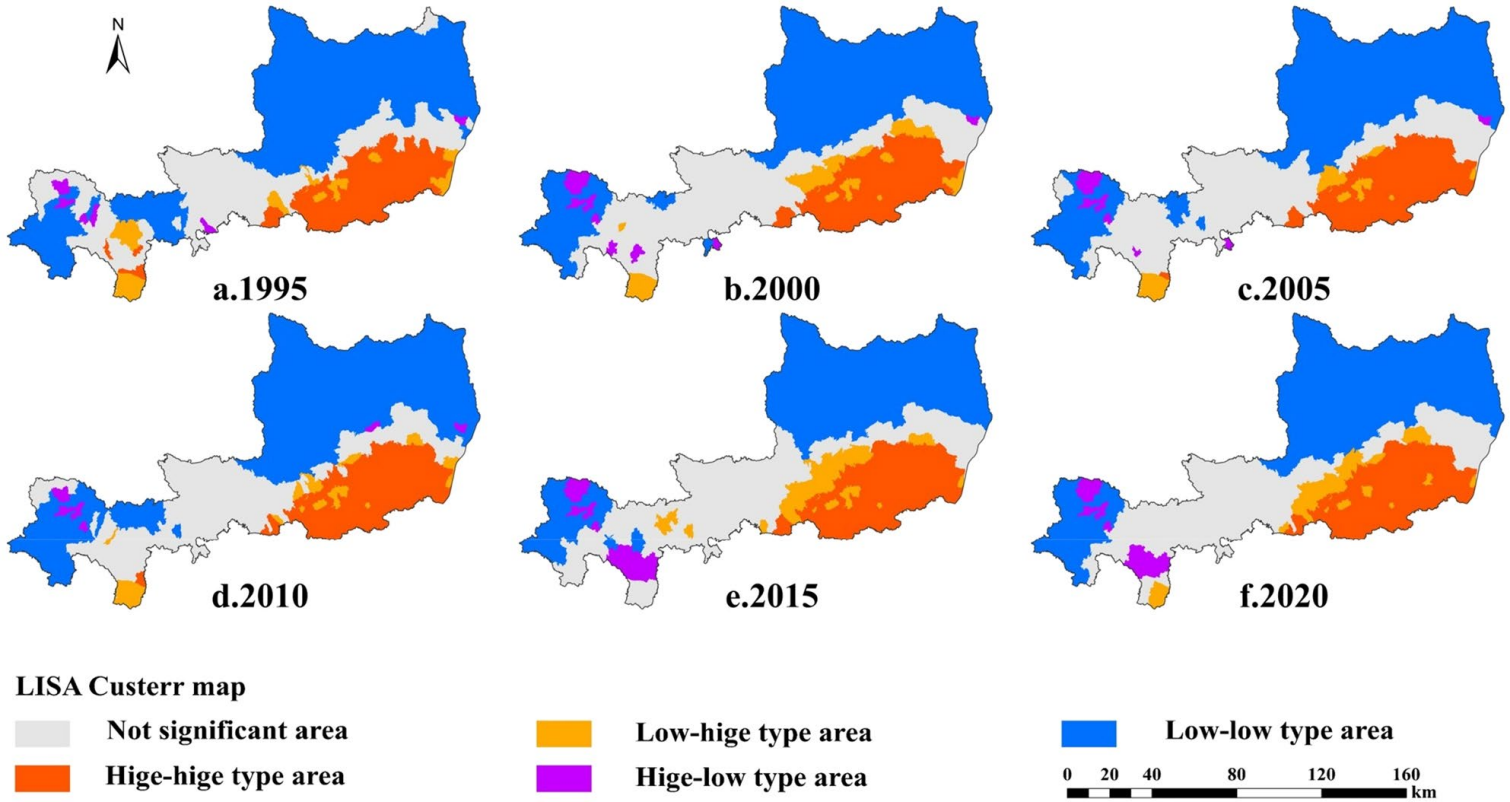
LISA plots for the areal proportion of arable land in Weibei dry plateau region during 1995–2020.

Using Geoda 1.14 software to draw the LISA agglomeration map (Fig. 7), based on the calculated results, combined with their spatial distribution status, the townships can be classified into the following four types.

1. "High-high type area": This area indicates that its township has a high proportion of arable land, accompanied by a high proportion of area in all its surrounding townships. This type of area has the most concentrated distribution of arable land, mainly in the southeast of the study area, which is a relatively flat plain area with a high level of agricultural development and a high proportion of arable land area.
2. "Low-low type area": This area indicates that its township has a low proportion of cultivated land, accompanied by a low proportion of cultivated land in all its surrounding townships. The distribution of cultivated land in this type of area is more concentrated, mainly in the northern and western high-altitude mountainous areas of the study area.
3. "Low–high type area": This type of area indicates that the township itself has a low proportion of cultivated land, but its surrounding neighboring areas have a high proportion of cultivated land. This type of area is more scattered, mainly distributed in the junction of the plateau gully area and the plain area in the study area. The main characteristics of the region are complex terrain, widely varying irrigation capacity and weak farming capacity.
4. "High-low type area": This type of area indicates that the township itself has a high proportion of arable land, but its neighboring areas have a low proportion of township area. This area is mainly located in the western part of the study area and is accompanied by the "low-low type area".

#### LISA frequency geo-spectrum analysis

**Figure 8:**
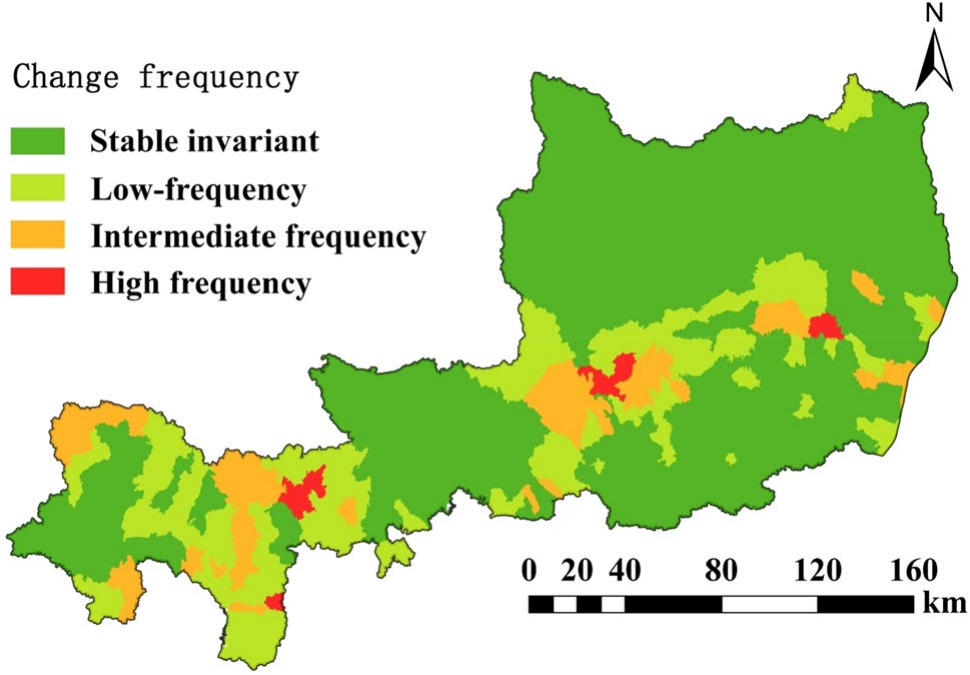
Analysis of LISA frequency geo-spectrum of cultivated land in the study area during 1995–2020.

Based on the LISA agglomeration map, LISA frequency mapping was constructed by counting the change frequencies of spatial units (Table 3, Fig. 8) to visually reflect the continuity and variability of spatial autocorrelation changes, which were analyzed as follows.

1. Stable invariant area: This type exhibits spatial units that are stable and unchanged for 25 years, accounting for 68.39% of the total area of the study area. The region is mainly composed of "high-high type areas", "low-low type areas" and insignificant areas. It is mainly distributed in the southern plains, the northern plateau gully area,and the central region of the study area.
2. Low-frequency area: This area consists of areas with 1 to 2 spatial aggregation transformations, accounting for 21.19% of the total area of the study area. It was mainly distributed at the intersection of high-high and low-low aggregation areas in the study area, and mostly showed low-low and insignificant interconversion.
3. Intermediate frequency area: This area is more scattered, accounting for 8.96% of the total area of the study area. The main manifestations are "low-low" aggregation and "low–high" aggregation to insignificant shift, distributed at the junction of low-frequency areas.
4. High frequency area: The High frequency area only accounts for 1.46% of the total area of the study area and is the most scattered. The number of transitions in this region is 4, the most active, mainly from the "low–high" aggregation area to the insignificant shift.

**Table 3:** Characteristics of LISA frequency geo-spectrum of cultivated land in the study area from 1995 to 2020.

Geo-spectrum of frequency	Times of spatial agglomeration changes	Area (km^2^)	(%)	Maximum map unit type
Stable invariant	0	27,192.49	68.39	NS, LL, HH
Low-frequency	1–2	8423.46	21.19	NS → LL, LL ← NS
Intermediate frequency	3	3562.46	8.96	LL, LH → NS
High frequency	4	580.74	1.46	LH → NS

### Analysis of transfer matrix of land use

As Table 4 demonstrates, a large number of cropland have been transformed into construction land during the 25 years, the transformed construction land have occupied a 43.09% (1017.26 km^2^). Meanwhile, some arable land was converted into forest land and grassland. By the amount of conversion, the area of forest land and grassland converted into arable land is larger, with 702.72 km^2^ and 605.11 km^2^ respectively, accounting for 73.76% of the total amount of conversion. Additionally, less variation in bare land and water in the study area. In general, arable land is mainly converted to construction land, and some arable land is converted to forest land and grassland. Also, forest land and grassland are also the main sources of conversion of arable land, and there is interconversion between them.

**Table 4:** Transfer matrix of land use in the study area from 1995 to 2020 km^2^.

Year	Item	Froestland	Grass land	Construction land	Bare land	Water	Total
1995–2020	Transfer out	775.82	484.69	1017.26	14.72	68.1	2360.59
	Transfer to	702.71	605.11	353.42	8.11	103.65	1773

### Analysis of the drivers of the spatial and temporal evolution of cropland

In this study, 15 potential factors were selected and a Geo-detector was used to analyze the drivers of arable land change in the Weibei Dry Plateau Region of Shaanxi from 2000 to 2020, thereby exploring the strength of individual influencing factors and factor interactions (Table 1). As an agricultural region, the total power of agricultural machinery and the increase in the secondary industry were specifically selected as additional driving factors in the selection of driving factors. In contrast to other studies, nighttime lighting data was selected as one of the driving factors. Referring to the method of Cao et al. (2013) for discrete classification of each driving factor, the natural breakpoint method in ArcGIS 10.6 was used to convert continuous data into categorical data and classify the influencing factors into 4–5 categories, taking into account the actual situation of the region. The details are shown in the attached table (1–1).

#### Analysis of factor detector

Factor detector was used to analyze natural and socioeconomic factors for three time periods (2000, 2010 and 2020) to detect the intensity of the effect of different factors on arable land expansion.

**Figure 9:**
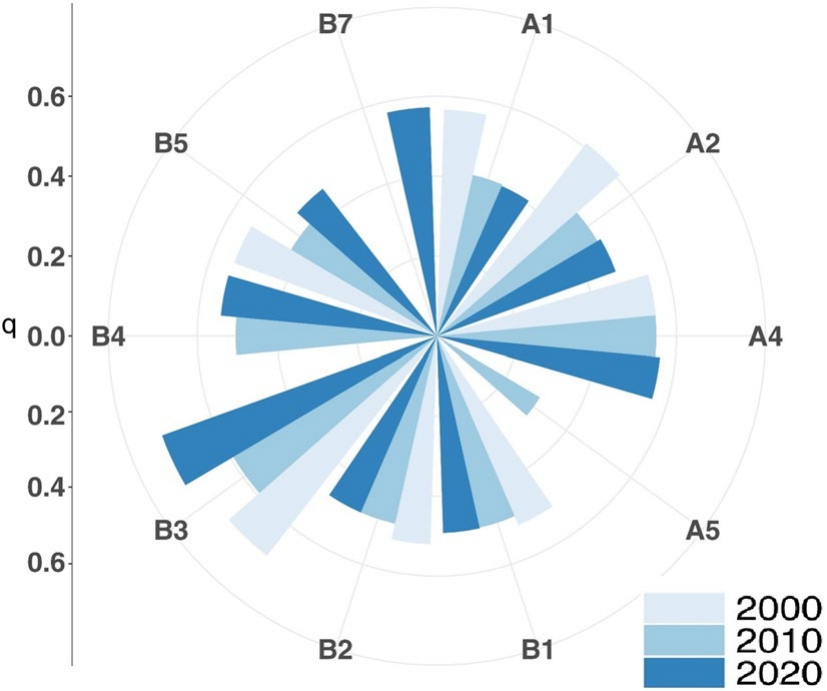
The q value of driving factors in different years in the study area.

A comparative analysis of the factors that passed the significance test shows that (Fig. 9): the explanatory power of the natural and socioeconomic factors on the change of cropland in the study area in 2000 ranged from 0.514 to 0.694, and the strength of the total power of agricultural machinery (0.694) was the largest; the strength of the factors on the change of cropland in 2010 ranged from 0.299 to 0.592, which was lower than that in 2000. In 2010, the intensity of each factor on the change of cropland ranged from 0.299 to 0.592, showing an overall decreasing trend compared with 2000, and the intensity of each driver in 2020 ranged from 0.408 to 0.731, with large differences in the intensity of each driver. In general, the intensity of the effect of factors influencing the arable land area in the study area varied in different periods, but certain patterns still existed: the q value of total agricultural machinery power was always the largest in the three periods, indicating that this factor contributed the most to the change of arable land. Temperature, total population, population density, slope, value added of primary industry and elevation were all explained to a stronger extent in different periods, but the overall intensity of the effect did not change much. Natural conditions were the main influencing factors for cropland in 2000, and the intensity of the role of slope and elevation ranked lower as time changed, while the intensity of the role of total food production in 2010 and GDP per capita in 2020 came to the fore and the explanatory power of socio-economic factors ranked higher. In recent years, frequent human activities have led to the increase of agricultural productivity, resulting in the weakening of the intensity of the role of natural factors on arable land change, and the socio-economic factors directly determine the spatial pattern of arable land change to a large extent^30,31^.

#### Analysis of interaction detector

**Figure 10:**
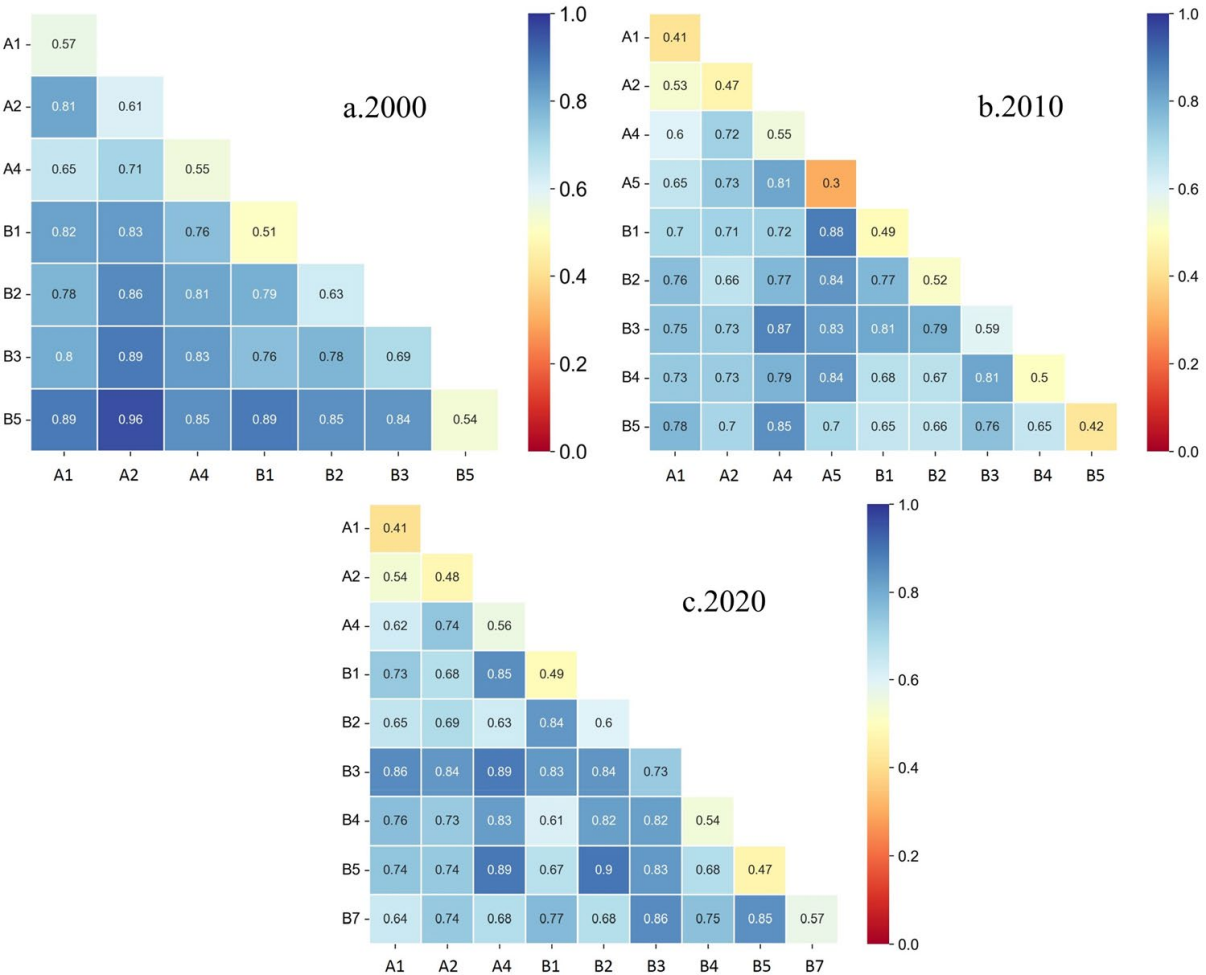
The interaction between natural and social economic factors in different periods in the study area.

The interaction between natural and socioeconomic factors was significantly greater than the interaction between single factors in the three periods (Fig. 10).Longitudinally, the compound influence of slope and each socio-economic factor in 2000 is higher, in descending order: slope and value added of primary industry (0.961), slope and total agricultural machinery power (0.893), slope and population density (0.858), and slope and total population (0.835); the compound influence of annual average temperature and each socio-economic factor in 2010 is the highest. The magnitude of the effect intensity ranged from 0.717 to 0.871; similarly, the compound influence of the annual average temperature in 2020 with each socioeconomic factor was the highest, and the effect intensity with the value added of primary industry was as high as 0.891, and the effect intensity with total agricultural machinery power, total population, and total grain production were 0.889, 0.855, and 0.826, respectively. The above results show that the main factors influencing the change of cropland are slope and average annual temperature. Besides, the factors affecting the change of cropland shifted from topographic factors such as slope to average annual temperature and average annual precipitation over time.

Cross-sectionally, the composite influence of total agricultural machinery power and value added of primary industry with each natural factor is higher, followed by the composite influence of total population and other factors with each natural factor. The intensity of the effect of each combination on the change of arable land in 2000 was above 70% (0.760–0.961), and the intensity of the effect of the primary industry and other factors was obvious; the interaction intensity decreased in 2010 (0.698–0.883), in which only the value added of the primary industry and the average annual temperature, the total power of agricultural machinery and the average annual temperature, the total power of agricultural machinery and the average annual precipitation had an effect on the intensity of the effect of change in arable land is higher than 0.8.The highest intensity of the effect of each factor on the change of arable land in 2020 reaches 0.889, and the same is shown by the highest intensity of the effect of total agricultural machinery power with each natural factor and the value added of primary industry with each natural factor, which are 0.857, 0.837, 0.889, 0.744, 0.738 and 0.826, respectively.

Throughout the study period, it can be found that the combined influence of natural and socio-economic factors on the change of cropland is significantly stronger than the combined influence between natural and social factors, and the interactive influence of natural and socio-economic factors leading to the change of cropland varies in different years, but the interactions all show a mutually reinforcing effect. In addition, the interaction between the factors brought out the influence of annual average temperature and value added of primary industry in 2000, 2010 and 2020.

## Discussion

### Spatial and temporal variation characteristics of arable land

The study adopted the center of gravity model and analyze the evolution characteristics of the spatial pattern of cropland by combining the total characteristics of cropland, the net change rate, the dynamic attitude of cropland change and standard deviational ellipse, which revealed the evolution characteristics of the spatial pattern of cropland in the Weibei Dry Plateau Region of Shaanxi Province from 1995 to 2020 in a more comprehensive and dynamic way, and mainly divided into three stages: (1) From 1995 to 2000, the cropland changed relatively slowly and the arable land area remained basically unchanged. The study area is located in a typical loess plateau gully area with complex and diverse topographic fragmentation, severe soil erosion, fragile ecological environment, and relatively poor agricultural production conditions^32^. This is consistent with previous studies. In addition, moisture is the main constraint to agricultural production in the Weibei Dry Plateau Region, and the agricultural economy develops more slowly due to the limitations of irrigation facilities and farming techniques, so the center of gravity of arable land moved less and more slowly. Wei Fang et al. found that it is moisture and topography that constrain agricultural development in the Weibei dry loess plateau area, which is largely consistent with the findings of that study^33^. (2) During 2000–2015, the arable land area shrank and the center of gravity of arable land shifted to northeast, which is in agreement with the results of previous studies. The main reason for the shrinkage of arable land is that with the development of urbanization and industrialization, high-quality arable land with good drainage and irrigation conditions around cities and villages has been heavily occupied or replaced. Cultivated land in the Manas Basin is likewise on a decreasing trend^34^; Beijing has experienced serious fragmentation of cultivated land^35^; pressure on cultivated land in central Hubei Province is increasing year by year as the population increases and demand for cultivated land output rises^36^; and Guizhou's karstic mountainous areas have also lost a large amount of arable land^37^, which is consistent with the results of this study.

Although the arable land area is decreasing, it can be seen by the shift of the center of gravity of arable land to the northeast: the arable land in the northeastern plateau region is slowly expanding due to the government's increased investment in agricultural modernization, the gradual improvement and renewal of agricultural infrastructure, and the popularization and improvement of new agricultural technologies. (3) From 2015 to 2020, the loss of arable land is the most serious, and the spatial distribution is more dispersed. The arable land area decreases by 293.96 km^2^ in only five years. In recent years, the rapid development of the regional economy, urban construction and development zone construction have occupied arable land resulting in the reduction of arable land area and also accelerated the rate of arable land loss^38–40^. This is consistent with previous findings.

### Land use change characteristics

The results of the land use transfer matrix indicate that the pressure of population growth may lead to the interconversion between arable land and forest and grassland, so that part of the barren grassland and forest land is reclaimed as arable land^41^. Wu Lina et al. showed that the same conversion of cropland-grassland to each other would exist in the Beiluo River Basin due to human activities^42^. Qiao Weifeng et al. showed that some forest-grassland would be converted to cropland in Suzhou City from 1999 to 2008. In the same year, the conversion of forest-grassland to cropland would occur in the Beiluo River Basin due to human activities^43^. Meanwhile, the central and local governments have adopted a series of measures to the way and extent of land use. For example, the project of returning arable land to forest and grass, which was fully launched in 2002, has transformed the arable land in the study area to forest land and grass, which can reduce soil erosion and effectively protect and improve the ecological environment. The conversion of arable land to construction land to a greater extent may be due to the expansion of construction land under rapid economic growth, improved living standards of residents and some industrial development, resulting in the occupation of arable land. This is consistent with previous findings^44^. In contrast, the conversion of construction land into arable land to a certain extent may be due to the following two reasons: first, the strict implementation of control documents such as "Opinions of the State Council of the CPC Central Committee on Strengthening the Protection of Arable Land and Improving the Balance of Occupancy and Replenishment" and "Notice on the Full Implementation of the Special Protection of Permanent Basic Farmland" issued by the state, which have played a protective role for arable land; second, the national land reclamation projects such as the reclamation of abandoned rural residential land and industrial land have regenerated and systematically restored industrial and mining land, etc., and to a certain extent, arable land has been replenished. Although the implementation of soil and water conservation projects in the country as well as in different regions has eased the rate of arable land reduction, the overall arable land area still shows a decrease^45^. Haikou City combined three periods of images, 1986, 1996, and 2000, to arrive at a large reduction in the area of cultivated land^46^.In the Yangtze River Basin, the decline in paddy and dry land reached 196.58% during the period 1980–2005^47^. In Inner Mongolia, cultivated land showed a decreasing trend from 1990–2018. In the same period, the decline in the area of cultivated land reached 196.58%^48^.The study shows that against the backdrop of decreasing arable land nationwide, arable land conservation in the Weibei Dry Plateau Region still needs to be strengthened. The implementation of land reclamation and ditch management in recent years has had a mitigating effect on the rapid decay of arable land. In addition, we should coordinate the relationship between urban development and arable land protection, vigorously carry out the construction of high-standard farmland, effectively protect high-quality arable land, and develop new types of arable land as soon as possible. Due to the complexity and variability of arable land changes, future studies such as arable land simulation and prediction are needed to better serve the conservation of arable land. However, the current application of the land use transfer matrix mainly focuses on the direct analysis of information on changes in the area of land use types, which is dominated by the analysis of net changes in the area of each land use type. This analytical model ignores the fixity and uniqueness of the spatial location of land use and does not consider the spatial process of land use dynamic change and related attributes, thus failing to fully cover and realistically portray the spatial process of land use change. In order to obtain systematic information on land-use change, it is necessary in the future to introduce into land-use change analysis the method of discovering superior information by comparing actual values with predicted values, so that land-use change information can be comprehensively obtained^49^.

### Drive analysis

In this study, the geo-detector is introduced to carry out the quantitative analysis of the driving forces of arable land change. The results show that total agricultural machinery power is always an important factor influencing the change of arable land in the study area. This is in line with previous findings^50^. and the degree of influence of total food production and GDP per capita has increased in the later years; meanwhile, the interactions among the factors show a two-factor enhancement in all years, indicating that the interactions among the factors have deepened their influence on natural and socio-economic factors, further promoting the change of arable land. It is noteworthy that the influence of social factors is becoming more and more significant. Compared with previous studies, this paper selects more influential factors for geodetector analysis and takes into account factors that respond to socioeconomic factors such as nighttime light brightness, contributing to a more comprehensive understanding of the driving forces affecting arable land change. With its unique advantage in spatial differentiation (Chen 2019), the geodetector is not only able to elaborate the intensity of the effect of arable land change in terms of a single factor, but also able to further explore the mechanism of land use change from the perspective of interaction using the interaction detector, which makes up for the shortcomings of conventional methods that can only analyze a single factor. In addition, this model eliminates the influence of covariance among factors, which is of practical significance for analyzing the change process of cropland from multiple perspectives.

In fact, traditional means of statistical or spatial analysis do not quantitatively give the extent to which influencing factors play a role in soil erosion, whereas the geoprobe approach can reveal the driving forces behind geographic phenomena by detecting their spatially stratified heterogeneity, with the core assumption that the spatial distributions of the independent and dependent variables are strongly consistent if a given independent variable has a significant effect on a given dependent variable. As there are still many scholars utilizing GWR for driver analysis at this stage, GWR explores the spatial variation of the research object and related drivers at a certain scale by establishing local regression equations at each point in the spatial scale and can be used for the prediction of future results. It has the advantage of higher accuracy because it takes into account the local effects of spatial objects. In the future, we need to deepen the analysis of the driving force of cropland change and compare the two methods.

## Conclusion

Based on six periods of cropland data from 1995 to 2020, this study integrates the center of gravity model, standard elliptical difference model and GIS technology to explore the trend of spatial pattern evolution in the Weibei Dry Plateau Region of Shaanxi Province. On this basis, the geodetector model is used to explore the intensity and mechanism of action of influencing factors affecting the change of cropland in different periods, and the following conclusions are drawn:

Over the past 25 years, the arable land area in the study area has been declining continuously, decreasing by 5.58% compared with 1995. The net loss of arable land is increasing, and the arable land is mainly converted into construction land, and the stability of arable land resources in the whole region is weakened. The problem of arable land loss in the northern plateau gullies has always been more prominent and the stability of arable land resources is poor.

Most of the regions in the study area show high (HH) or low (LL) clustering of cropland area ratios. The combined proportion of stable and constant and low-frequency areas in the LISA frequency mapping reaches 89.58%, and the spatial pattern of cultivated land is relatively stable. The mode of transformation in the middle and high frequency zones is mainly in the form of "low-low" aggregation, "low–high" aggregation transformation is not significant, and the attenuation of some arable land is more obvious.

From 1995 to 2020, the center of gravity of cropland in the study area shifted in the same direction as the standard ellipse, mainly to the northeast. The center of gravity of cropland shifts slowly (1995–2000) to accelerate (2000–2020), the ellipse area increases, and the spatial distribution of cropland tends to be dispersed.

Factor detection found that the total power of agricultural machinery had the strongest explanatory power, and the strength of the socioeconomic factors was accentuated over time. The results of the interaction detection showed that the strength of the inter-factor effect was greater than the interaction between single factors, both showing a two-factor enhancement. The inter-factor interaction brought out the influence of average annual temperature and value added of primary industry.

In general, against the backdrop of the nationwide decline in arable land, although the Weibei Dry Plateau region has actively carried out arable land conservation projects such as land reclamation and ditching and land reclamation, which have been effective in mitigating the trend of its decline, the conservation of arable land in the region will need to be continuously strengthened in the future.

### Supplementary Information

Supplementary Information.

## Data Availability

The datasets generated and analysed during the current study are not publicly available due this experiment was a collaborative effort, the trial data does not belong to me alone but are available from the corresponding author on reasonable request.

## References

[1] Wilken F, Wagner PD, Narasimhan B, Fiener P (2017). Spatio-temporal patterns of land use and cropping frequency in a tropical catchment of south india. Appl. Geogr..

[2] Ma J, Zhang C, Yun W, Lv Y, Zhu D (2020). The temporal analysis of regional cultivated land productivity with GPP based on 2000–2018 modis data. Sustainability.

[3] Wang YQ, Jiang-Ming MA, Yu-Ting LI, Jian R, Song ZR (2019). Spatio-temporal changes of land use in guangxi section of the pearl river-west river economic belt during 1980–2015. Environ. Ecol...

[4] Wang, L. Fractal characteristic analysis of urban land-cover spatial patterns with spatiotemporal remote sensing images in Shenzhen city (1988–2015). *Remote Sensing*. **13** (2021).

[5] Schillaci C, Acutis M, Lombardo L, Lipani A, Fantappiè M, Mrker M (2017). Spatio-temporal topsoil organic carbon mapping of a semi-arid mediterranean region: the role of land use, soil texture, topographic indices and the influence of remote sensing data to modelling. Sci. Total Environ..

[6] Li, Y., Chang, C., Zhao, Y., Wang, Z., Li, T., Li, J., et al. Evaluation system transformation of multi-scale cultivated land quality and analysis of its spatio-temporal variability. *Sustainability*, **13** (2021).

[7] Yang J, Wang J, Xu C, Liao X, Tao H (2022). Modeling the spatial relationship between rice cadmium and soil properties at a regional scale considering confounding effects and spatial heterogeneity. Chemosphere.

[8] Zhu Z, Liu B, Wang H, Hu M (2021). Analysis of the spatiotemporal changes in watershed landscape pattern and its influencing factors in rapidly urbanizing areas using satellite data. Remote Sensing.

[9] Fu, J., Zhang, Q., Wang, P., Zhang, L., Tian, Y., Li, X. Spatio-temporal changes in ecosystem service value and its coordinated development with economy: a case study in Hainan province, china. *Remote Sensing*, **14** (2022).

[10] Wang S, Zhang L, Zhang H, Han X, Zhang L (2020). Spatial–temporal wetland landcover changes of Poyang lake derived from landsat and hj-1a/b data in the dry season from 1973–2019. Remote Sensing.

[11] Yla, B., Xtcd, E., Rui, L., Sla, B., Avz, F., Key driving factors of selenium-enriched soil in the low-se geological belt: a case study in red beds of sichuan basin, china - sciencedirect. *Catena*, **196** (2021).

[12] Liu X, Song H, Lei T, Liu P, Zhao H (2020). Effects of natural and anthropogenic factors and their interactions on dust events in northern china. CATENA.

[13] Liu H, Qu M, Chen J, Xu G, Zhang J, Liu M (2022). Heavy metal accumulation in the surrounding areas affected by mining in china: spatial distribution patterns, risk assessment, and influencing factors. Science of The Total Environmen.

[14] Yong, Y.A., Xue, Y.A., Mh, B., Gcb, C., Beyond mere pollution source identification: determination of land covers emitting soil heavy metals by combining pca/apcs, geodetector and gis analysis. *CATENA*, **185** (2020).

[15] Shen Z, Zhang W, Peng H, Xu G, Chen X, Zhang X (2022). Spatial characteristics of nutrient budget on town scale in the three gorges reservoir area, china. Sci. Total Environ..

[16] Liu J, Xu Q, Yi J, Huang X (2022). Analysis of the heterogeneity of urban expansion landscape patterns and driving factors based on a combined multi-order adjacency index and geodetector model. Ecol. Indicators.

[17] Shi T, Hu Z, Shi Z, Guo L, Chen Y, Li Q (2018). Geo-detection of factors controlling spatial patterns of heavy metals in urban topsoil using multi-source data. Sci. Total Environ..

[18] Hong, H.A., Cs, B., Spatiotemporal variation and influencing factors of vegetation dynamics based on geodetector: a case study of the northwestern yunnan plateau, china. *Ecol. Indicators*, **130** (2022).

[19] Ren, D., Envelope, A., Analysis of the heterogeneity of landscape risk evolution and driving factors based on a combined geoda and geodetector model. *Ecol. Indicators*, **144** (2021).

[20] Su Y, Li T, Cheng S, Wang X (2020). Spatial distribution exploration and driving factor identification for soil salinisation based on geodetector models in coastal area. Ecol. Eng..

[21] Sun D, Shi S, Wen H, Xu J, Wu J (2021). A hybrid optimization method of factor screening predicated on geodetector and random forest for landslide susceptibility mapping. Geomorphology.

[22] Li, Y. Quantitative assessment of landslide risk based on susceptibility mapping using random forest and geodetector. *Remote Sensing*, **13** (2021).

[23] Hu Y, Yao Y, Kou Z (2021). Correction: exploring on the climate regionalization of qinling-daba mountains based on geodetector-svm model. PLoS ONE.

[24] Yca, B., Hl, A., Hk, A., Ml, A., Qin, F.A., Zx, C. Spatio-temporal variation of ozone pollution risk and its influencing factors in china based on geodetector and geospatial models. *Chemosphere* **32** (2021). 10.1016/j.chemosphere.2022.134843 35533939

[25] Wang, J. Analysis of differences in the spatial distribution among terrestrial mammals using geodetector—a case study of china. *ISPRS Int. J. Geo-Inf.*, **10** (2021).

[26] Zeng W, Wan X, Lei M, Gu G, Chen T (2022). Influencing factors and prediction of arsenic concentration in pteris vittata: a combination of geodetector and empirical models. Environ. Pollut..

[27] Chen, X. Quantifying influences of natural and anthropogenic factors on vegetation changes based on Geodetector: a case study in the Poyang lake basin, china. R*emote Sens.*, **13** (2021).

[28] Wang, J., Xu, C. Geodetector: principle and prospective. *Acta Geographica Sinica* 2017.

[29] Zhu GY, Shangguan ZP, Deng L (2021). Variations in soil aggregate stability due to land use changes from agricultural land on the loess plateau, china. CATENA.

[30] Han C, Shen Y, Lanzhen WU, Guo Y, Chen X (2021). Spatial and temporal variation characteristics of cultivated land in the upper yellow river from 2002 to 2018 based on time series modis. Chin. J. Eco-Agric..

[31] Wang H, Zhu Y, Wang J, Han H, Niu J, Chen X (2022). Modeling of spatial pattern and influencing factors of cultivated land quality in henan province based on spatial big data. PLoS ONE.

[32] Jin H, Shi D, Lou Y (2021). Evaluation of the quality of cultivated-layer soil based on different degrees of erosion in sloping farmland with purple soil in China. Catena.

[33] Yu F, Liu J, Xia L (2022). Landscape ecological risk assessment based on LUCC in the Weibei dry loess plateau area of Shaanxi. China Environ. Sci..

[34] Zhang Y, Zhou Z, Huang D (2020). Spatio-temporal evolution of cultivated land and analysis of influence factors in karst mountainous areas. Trans. Chinese Soc. Agric. Eng..

[35] Li C, Zhang F, Zhu T (2013). Analysis on spatial-temporal heterogeneities of landscape fragmentation in urban fringe area: A case study in Shunyi district of Beijing. Acta Ecol. Sinica.

[36] Lu X, Liu R, Kuang B (2022). Regional differences and dynamic evolution of cultivated land pressure in Hubei Province. Trans. Chinese Soc. Agric. Eng..

[37] Yang Q, Bi G, Chen Z (2018). Spatial allocation of fallow land in karst rocky desertification areas: A case study in Qinglong County, Guizhou Province. Acta Geogr. Sinica.

[38] Calzolari C, Ungaro F, Vacca A (2021). Effectiveness of a soil mapping geomatic approach to predict the spatial distribution of soil types and their properties. CATENA.

[39] Lin F, Chen X, Yao H, Lin F (2022). Swat model-based quantification of the impact of land-use change on forest-regulated water flow. CATENA.

[40] Yao Z, Zhang L, Tang S (2017). The basic characteristics and spatial patterns of global cultivated land change since the 1980s. J. Geogr. Sci..

[41] Min M, Duan X, Yan W, Miao C (2022). Quantitative simulation of the relationships between cultivated land-use patterns and non-point source pollutant loads at a township scale in chaohu lake basin, china. CATENA.

[42] Wu L, Yang S, Liu X (2014). Response of land use change to the degree of human activities in the Beiluo River Basin since 1976. J. Geogr..

[43] Qiao W, Sheng Y, Fang B (2014). Mining information on land use evolution in highly urbanized areas based on transfer matrix–Taking Suzhou City, Jiangsu Province as an example. Geogr. Res..

[44] Wang YH, Dai E, Yin L (2018). Land use/land cover change and the effects on ecosystem services in the Hengduan Mountain region, China. Ecosyst. Serv..

[45] Chen L, Cai H, Zhang T, Zhang X, Zeng H (2022). Land use multi-scenario simulation analysis of rao river basin based on markov-flus model. Acta Ecol. Sinica.

[46] Tian G, Zhang Z, Wang C (2001). Study on the dynamic change of land use structure in Haikou City based on remote sensing and GIS. J. Natl. Resour..

[47] Xu S, Zhang Y, Du M (2017). Spatio-temporal change characteristics of land use and its runoff effect in the Yangtze River Basin. Adv. Geosci..

[48] Wang N, Yang G, Han XY (2020). Land use change and ecosystem service value in Inner Mongolia from 1990 to 2018. J. Soil Water Conserv..

[49] Pontius RG, Shusas E, McEachern M (2004). Detecting important categorical land changes while accounting for persistence. Agric. Ecosyst. Environ..

[50] Tian GJ, Duan JL, Yang L (2021). Spatio-temporal pattern and driving mechanisms of cropland circulation in China. Land Use Policy.

