# Supplementary material for: Analysis of spatial and temporal changes and driving forces of arable land in the Weibei dry plateau region in China
Source: Sci Rep. 2023 Nov 23;13:20618. doi: 10.1038/s41598-023-43822-3 (PMC10667363; doi:10.1038/s41598-023-43822-3)
Supplement: Supplementary file 1 — Supplementary Information. [file 41598_2023_43822_MOESM1_ESM.docx]

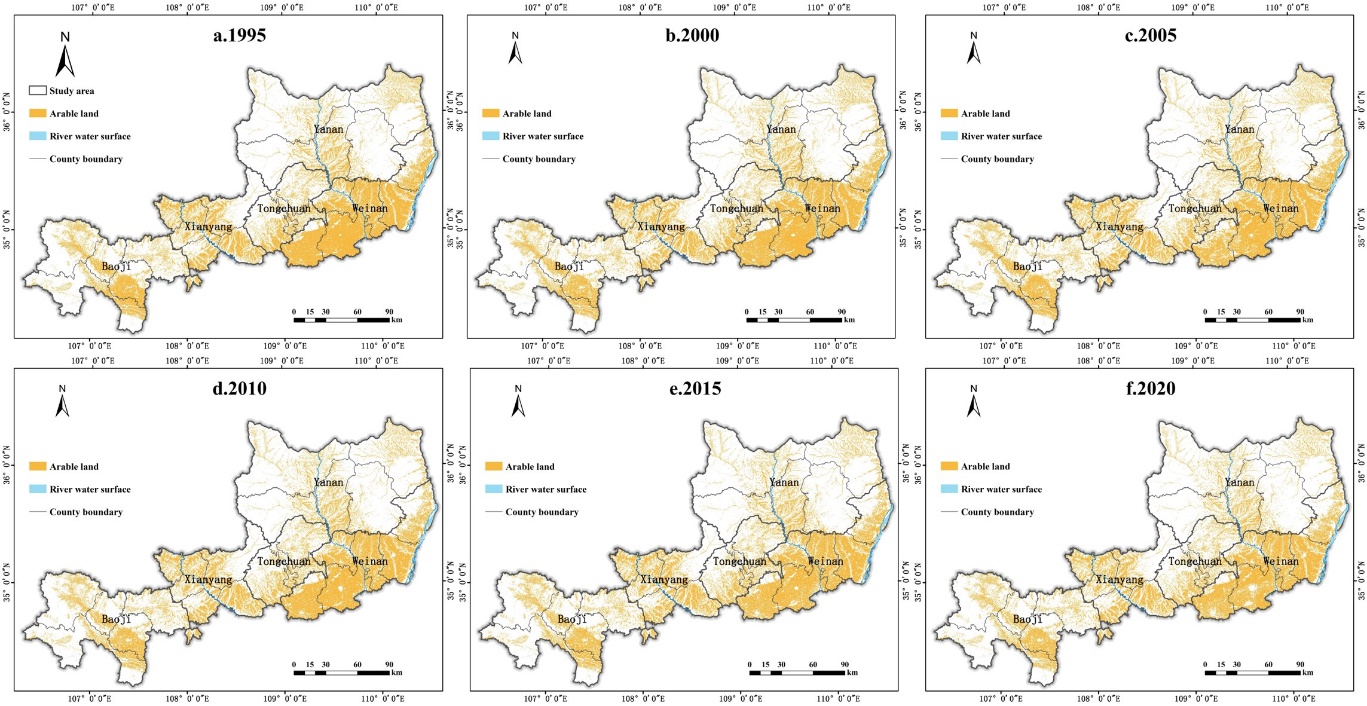


**Attachment 1** Arable land area in Weibei Dry Plateau Region

**Attachment 1** Driving factors affecting arable land changes in Weibei Dry Plateau Region

|  | Element | Code | Factor | Factor meaning |
| --- | --- | --- | --- | --- |
| Natural factors | Topography | A1 | Elevation/(m) | Elevation of the study area |
|  |  | A2 | Slope/(°) | Slope of the study area |
|  |  | A3 | Type of landform | Landform type in the study area |
|  | Climate | A4 | Temperature/(°C) | Average annual temperature |
|  |  | A5 | Precipitation/(mm) | Average annual precipitation |
|  |  | A6 | Soil type | Soil type in the study area |
| Socioeconomic factors | Population distribution | B1 | Total population/(million) | Total population by county |
|  |  | B2 | Population density | Total population density by county |
|  | Agricultural scale | B3 | Total power of agricultural  machinery/(million kilowatts) | Total agricultural machinery power by county |
|  |  | B4 | Total grain output/(ton) | Total grain production by county |
|  |  | B5 | Increase in primary industry  /(million yuan) | Increase in primary industry by county |
|  |  | B6 | Increase in secondary industry  /(million yuan) | Increase in secondary industry by county |
|  | Economic level | B7 | GDP per capita/(yuan) | GDP per capita by county |
|  |  | B8 | Night lighting brightness | Night light brightness distribution |
|  |  | B9 | Fixed asset investment  /(billion yuan) | Fixed asset investment amount by county |

**Attachment 2** Stratification of different factors in GDM of 2000

| Impact  factions | Stratification | | | | |
| --- | --- | --- | --- | --- | --- |
|  | 1 | 2 | 3 | 4 | 5 |
| A1 | 506.66-641.45 | 641.45-834.52 | 834.52-1136.89 | 1136.89-1275.32 | 1275.32-1435.61 |
| A2 | 3.60-4.88 | 4.88-8.16 | 8.16-12.01 | 12.01-13.33 | 13.33-16.67 |
| A3 | ST1 | ST2 | ST3 | - |  |
| A4 | 93.40-100.56 | 100.56-107.19 | 107.19-115.92 | 115.92-127.27 | 127.27-137.92 |
| A5 | 5154.25-5302.98 | 5302.98-5471.54 | 5471.54-5729.34 | 5729.34-5997.05 | - |
| A6 | LT1 | LT2 | LT3 | LT4 | - |
| B1 | 4.6-17.04 | 17.04-31.14 | 31.14-49.66 | 49.66-75.1 | - |
| B2 | 16.78-59.14 | 59.14-191.50 | 191.50-339.75 | 339.75-604.47 | 604.47-1 366.88 |
| B3 | 0.5-2.9 | 2.9-6.5 | 6.53-12.30 | 12.30-34.7 | - |
| B4 | 12050-44397 | 44397-65643 | 65643-121115 | 121115-317295 | - |
| B5 | 2400-9952 | 9952-18316 | 18316-25663 | 25663-40962 | 40962-69190 |
| B6 | 1731-17386 | 17386-38640 | 38640-55118 | 55118-107411 | - |
| B7 | 1281-1587.21 | 1587.21-1874.30 | 1874.30-2218.79 | 2218.79-2984.34 | 2984.34-6161.37 |
| B8 | 0.06-0.54 | 0.54-1.13 | 1.13-2.01 | 2.01-4.10 | 4.10-9.44 |
| B9 | 608-2504 | 2504-5229 | 5229-8700 | 8700-12000 | 12000-51635 |

**Attachment 3** Stratification of different factors in GDM of 2010

| Impact  factions | Stratification | | | | |
| --- | --- | --- | --- | --- | --- |
|  | 1 | 2 | 3 | 4 | 5 |
| A1 | 506.66-834.52 | 834.52-1136.89 | 1136.89-1275.32 | 1275.32-1435.61 | - |
| A2 | 3.60-8.16 | 8.16-12.01 | 12.01-13.33 | 13.33-16.67 | - |
| A3 | ST1 | ST2 | ST3 | - | - |
| A4 | 97.25-103.94 | 103.94-110.62 | 110.62-120.13 | 120.13-142.12 | - |
| A5 | 5495.68-6030.02 | 6030.02-6414.75 | 6414.75-6934.84 | 6934.84-7312.45 | - |
| A6 | LT1 | LT2 | LT3 | LT4 | - |
| B1 | 5.15-14.90 | 14.90-31.82 | 31.82-51.90 | 51.90-78.29 | - |
| B2 | 18.80-60.08 | 60.08-158.12 | 158.12-297.44 | 297.44-632.84 | 632.84-1334.60 |
| B3 | 3-6 | 6-11 | 11-17 | 17-33 | 33-59 |
| B4 | 1028-5028 | 5028-7949 | 7949-18245 | 18245-41388 | - |
| B5 | 5700-36990 | 36990-5033 | 50339-9714 | 97143-141920 | - |
| B6 | 3840-90530 | 90530-287060 | 287060-580600 | 580600-841790 | - |
| B7 | 6387.15-9082.01 | 9082.01-12252.42 | 12252.42-30482.30 | 30482.30-46809.94 | - |
| B8 | 0.24-2.73 | 2.73-4.96 | 4.96-8.59 | 8.59-16.5 | - |
| B9 | 30538-105880 | 105880-213752 | 213752-300372 | 300372-450090 | 450090-660897 |

**Attachment 4** Stratification of different factors in GDM of 2020

| Impact  factions | Stratification | | | | |
| --- | --- | --- | --- | --- | --- |
|  | 1 | 2 | 3 | 4 | 5 |
| A1 | 506.66-834.52 | 834.52-1136.89 | 1136.89-1275.32 | 1275.32-1435.61 | - |
| A2 | 3.60-4.88 | 4.88-8.16 | 8.16-12.01 | 12.01-13.33 | 13.33-16.67 |
| A3 | ST1 | ST2 | ST3 | - | - |
| A4 | 98.38-105.78 | 105.78-113.56 | 113.56-117.81 | 117.81-128.73 | 128.73-145.57 |
| A5 | 4222.31-4755.22 | 4755.22-5295.86 | 5295.86-5859.66 | 5859.66-6191.77 | - |
| A6 | LT1 | LT2 | LT3 | LT4 | - |
| B1 | 4.87-13.35 | 13.35-21.83 | 21.83-44.64 | 44.64-79.44 | - |
| B2 | 1022.37-1515.17 | 1515.17-3363.17 | 3363.17-5087.98 | 5087.98-7305.58 | - |
| B3 | 4-9 | 9-15 | 15-30 | 30-41 | 41-72 |
| B4 | 10363-5670 | 5670-9057 | 9057-17555 | 17555-36020 | - |
| B5 | 1290-7965 | 7965-15978 | 15978-21295 | 21295-29288 | - |
| B6 | 1066-13770 | 13770-41830 | 41830-76023 | 76023-142710 | 142710-231781 |
| B7 | 23154.68-32500.77 | 32500.77-42946.41 | 42946.41-63287.92 | 63287.92-111667.72 | 111667.72-163346.15 |
| B8 | 2.72-5.45 | 5.45-7.33 | 7.33-8.48 | 8.48-18.19 | - |
| B9 | 166004-576343 | 576343-978927 | 978927-1559400 | 1559400-2112177 | 2112177-4067211 |

ST1: yellow cotton soil; ST2: brown soil; ST3: black kiln soil; LT1; river valley and riverine land; LT2; loess mountain plateau: LT3; loess plateau: LT4: middle and low mountainous land
